# Post-Treatment Thyroid Diseases in Children with Brain Tumors: A Single-Center Experience at “Prof. Dr. Ion Chiricuță” Institute of Oncology, Cluj-Napoca

**DOI:** 10.3390/diagnostics10030142

**Published:** 2020-03-05

**Authors:** Maria Margareta Cosnarovici, Andra Piciu, Eduard-Alexandru Bonci, Marius-Ioan Bădan, Claudiu-Iulian Bădulescu, Andreea-Ioana Stefan, Alexandru Mester, Rodica Cosnarovici, Maria-Iulia Larg

**Affiliations:** 1 Department of Medical Oncology Iuliu Hațieganu, University of Medicine and Pharmacy, 400012 Cluj-Napoca, Romania; cosnarovici_maria@yahoo.com; 2 Department of Surgical Oncology Iuliu Hațieganu, University of Medicine and Pharmacy, 400012 Cluj-Napoca, Romania; bonci.eduard@gmail.com; 3 Department of Anatomy and Pathology Iuliu Hațieganu, University of Medicine and Pharmacy, 400012 Cluj-Napoca, Romania; badan_marius@yahoo.com (M.-I.B.); claubad05@yahoo.com (C.-I.B.); 4 2nd Pediatric Departament Iuliu Hațieganu, University of Medicine and Pharmacy, 400012 Cluj-Napoca, Romania; andreea.stefan24@gmail.com; 5 Department of Oral Health Iuliu Hațieganu, University of Medicine and Pharmacy, 400012 Cluj-Napoca, Romania; alexandrumester@yahoo.com; 6 Department of Pediatric Oncology “Prof.Dr.IonChiricuță”, Institute of Oncology, 400012 Cluj-Napoca; Romania; rodicacosnarovici@yahoo.com; 7 PhD School Iuliu Hațieganu, University of Medicine and Pharmacy, 400012 Cluj-Napoca, Romania; mariaiulia.larg@yahoo.com

**Keywords:** children, thyroid dysfunction, brain tumors, follow-up

## Abstract

Aim of study: The purpose of the study was to evaluate the association of thyroid dysfunction occurring in pediatric patients treated for brain tumors. Patients and methods: A total of 255 patients with brain tumors were treated between 2001 and 2018 at the “Prof. Dr. Ion Chiricuță” Institute of Oncology, Cluj-Napoca. Due to a minimum follow-up of 4 years, we studied 184 out of the 255 patients. The cohort included 69 girls (37.5%) and 109 boys (62.5%), with a median age of 8.4 years. The evaluated tumors included medulloblastomas (47 patients), astrocytomas (44 patients), ependymomas (22 patients), gliomas (20 patients), germ cell tumors (12 patients), primitive neuroectodermal tumors (4 patients), as well as other types of tumors (15 patients); in 20 of the cases, biopsy could not be performed. Results: There was a 60% overall survival rate; among the 120 surviving patients, 11 (9.1%) were diagnosed with iatrogenic thyroid disease. We observed an important number of iatrogenic thyroid disease cases in this group of patients, thus revealing the importance of long-term thyroid function evaluation in all children who finalized their treatment for brain tumors. Through this study, we aimed to provide an accurate image of the methodology of monitoring for thyroid dysfunction in childhood brain tumor survivors. Conclusion: Given the fact that the probability of developing thyroid dysfunction in the pediatric population treated for brain tumors is not rare, we recommend that childhood brain tumor survivors be monitored for iatrogenic thyroid disease, in order to provide early diagnosis and treatment.

## 1. Introduction

The tumors of the central nervous system, which are represented by histologically diverse neoplasms, represent the second most frequent type of cancer in children after leukemia. Childhood brain tumors have a relatively poor survival rate compared to other childhood malignancies; this is why efforts to develop new diagnostic and therapeutic tools have greatly increased in recent years. As a result of the development of these new diagnostic and treatment tools, an increasing number of long-term survivors has been registered worldwide, revealing an important number of late side effects. Quality of life in this patient group has become a major field of interest, demonstrating the need for new and improved techniques for managing neurologic, sensory, and endocrinologic deficiencies. Childhood brain tumor survivors, especially those who received radiation therapy to the craniospinal axis, are at risk of developing endocrine deficiencies [1].

The number of long-term survivors of childhood brain tumors has grown compared to the previous decade, and it has become clear that the new tools of oncological treatment may have serious long-term effects. The current focus on diagnosing and managing neurologic and sensory outcomes has proven its utility in improving quality of life in this group of patients. 

Thyroid dysfunction is the most common endocrine complication in patients treated for childhood brain tumors worldwide, thus revealing the importance of frequent monitoring of their thyroid function [2,3]. Permanent long-term thyroid dysfunction is most frequent following high-dose irradiation to the hypothalamic and suprasellar regions. Radiation therapy of the posterior fossa is less likely to cause thyroid malfunctions; however, doses higher than 50 Gy administered in this region often produce hearing impairments [4]. More than 20% of the patients receiving frontal lobe radiation develop motor impairments. Any cortical segment of the brain receiving more than 30 Gy is at risk of becoming a focal site for seizure development [5]. None of the standard chemotherapy regimens used for treating childhood brain tumors are known to cause thyroid deficiencies [6].

Knowing that thyroid hormone is essential to growth and neurologic development during childhood, diagnosing and treating thyroid malfunctions are both essential in order to assure normal growth and avoid developmental delay [7]. Central hypothyroidism due to hypopituitarism or hypothalamic lesion leads to hypothyroidism, which has subtle clinical features. The common thyroid function test results for central hypothyroidism include low or normal serum thyroid-stimulating hormone (TSH) and low serum free thyroxine (FT4). The difference between secondary and tertiary hypothyroidism is established by performing a thyrotropin-releasing hormone (TRH) stimulation test. If there is no change in the serum TSH values following the administration of TRH, the test is interpreted as secondary hypothyroidism; in hypothalamic (tertiary) hypothyroidism, TSH values increase [8].

The role of this study was to propose a protocol of thyroid follow-up in the monitoring of childhood brain tumor survivors, in order to provide early diagnosis of thyroid gland dysfunction.

## 2. Patients and Methods

A total of 255 children diagnosed with primary brain tumors received treatment at the “Prof. Dr. Ion Chiricuță” Institute of Oncology, Cluj-Napoca between 2001 and 2018. Due to a minimum follow-up of 4 years, we studied 184 patients out of the 255. Six patients abandoned the therapy (2.71%). The gender distribution was 69 girls (37.5%) and 109 boys (62.5%), with a median age of 8.4 years (0.2–17.9 years), and the median follow-up was 71.4 months (1–227 months). Death occurred in 64 (34.8%) cases.

For all children, informed consent was legally obtained and signed by the parents, and the study was approved by the Ethics Committee of the Institute of Oncology, Cluj-Napoca (No 165/28.11.2019). The evaluated tumors included medulloblastomas (47 patients, 25.5%), astrocytomas (44 patients, 23.9%), ependymomas (22 patients, 11.9%), gliomas (20 patients, 10.9%), germ cell tumors (12 patients, 6.5%), and primitive neuroectodermal tumors (4 patients, 2.2%); 15 patients (8.2%) were diagnosed with other types of tumors, and in 20 patients (10.9%), a biopsy could not be performed due to the tumor location. Regarding the morphological aspect of the 44 astrocytomas, we recorded Grade 1 (26 patients), Grade 2 (11 patients), and high-grade tumors (7 patients). The 20 gliomas were either low-grade optic nerve glioma (6 patients) or glioblastoma multiforme (14 patients). All patients in this retrospective cohort were diagnosed according to the World Health Organization (WHO) 2001 Classification of Primary Brain Tumors.

In all 178 patients, therapeutic response was assessed according to the “Response Evaluation Criteria for Solid Tumors” (RECIST) that was in use at the time of the treatment. Complete remission was defined as the disappearance of all target lesions. Partial remission was defined according to the RECIST in use at the time of treatment.

The first therapeutic act was surgery for all the operable tumors. One hundred and fifty-eight patients underwent surgery; of these children, we registered complete remission in 48.1% (76 patients) and partial remission in 51.9% of the cases (82 patients). Twenty patients were diagnosed with inoperable tumors, leading to poor therapeutic results; we registered 1 case of complete remission (5%) and 19 cases of partial remission (95%). One hundred and twenty patients received radiation to the craniospinal axis (i.e., whole brain, posterior fossa, and spinal cord) and fifty-eight did not receive radiation. Children under 3 years of age did not receive radiation therapy.

The chemotherapy regimens consisted of temozolomide, carmustine, lomustine cisplatin, carboplatin, etoposide, irinotecan, methotrexate, cytosine-arabinoside, procarbazine, vincristine, cyclophosphamide, and ifosfamide.

A total of 133 patients received standard chemotherapy, while 45 patients did not undergo chemotherapy. Of these children, we registered complete remission in 43.25% of the cases (77 patients) and partial remission in 56.74% of the cases (101 patients).

## 3. Results

**Figure 1:**
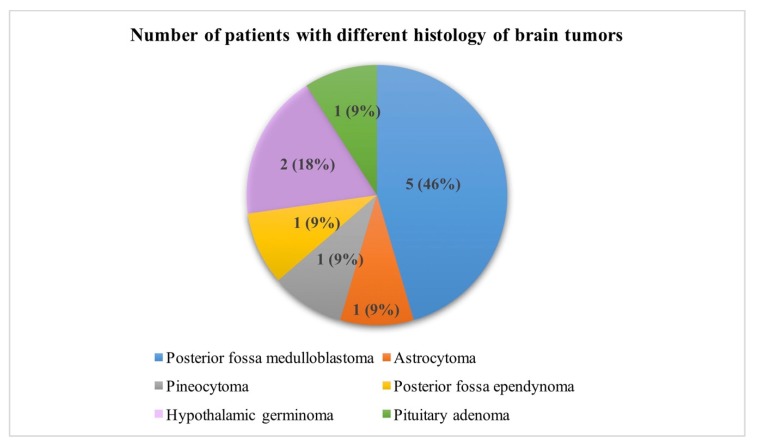
Histology of brain tumors in patients with iatrogenic thyroid disease.

A total of 255 patients with brain tumors were treated between 2001 and 2018 at the “Prof. Dr. Ion Chiricuță” Institute of Oncology, Cluj-Napoca. Due to a minimum follow-up of 4 years, we studied 184 out of the 255 patients. Six patients abandoned the therapy at different stages of treatment. One hundred and twenty patients (65.20%) were alive at an interval of 4 years post-treatment. Of these patients, 11 (9.1%) were diagnosed with iatrogenic thyroid disease. The histology of the 11 children who developed iatrogenic thyroid disease is presented in Figure 1. Nine out of the eleven cases were high-grade tumors. The children’s ages at the time of treatment varied from 6 to 16 years, with a median age of 8.14 years. 

All eleven patients underwent surgery as follows: total surgery (seven patients), subtotal surgery (two patients), and biopsy (two patients); radiotherapy of the whole brain and/or spinal cord was universally employed in their treatment; and all high-grade tumors received standard chemotherapy. Four patients received radiation of the whole craniospinal axis, with doses ranging from 40 to 68 Gy to the tumor and from 22.4 to 36 Gy to the whole brain and spinal cord. Seven patients received brain radiotherapy alone. The radiation doses to these tumors ranged from 45 to 54 Gy (Table 1).

Four out of the eleven patients had documented thyroid dysfunction from the beginning of treatment. In these patients, the tumors were located close to the hypothalamic–pituitary axis. The histology was represented by germinoma in two cases, low-grade astrocytoma in one case, and pituitary adenoma for the other case. In all four cases, the patients developed combined pituitary hormone deficiency as a result of tumoral growth and compression of the hypothalamic–pituitary axis. In three of the cases, hypopituitarism was characterized by multiple pituitary hormone deficiencies, including thyroid-releasing hormone, somatotroph hormone, adrenocorticotropic hormone, and antidiuretic hormone. In the other case, the patient was diagnosed with hypothyroidism and diabetes insipidus. All these deficiencies persisted throughout treatment and caused important difficulties in therapeutic planning and management. All four patients have been found to be in long-term complete remission, with event-free intervals ranging from 6 to 11 years. The endocrinologic deficiencies have remained identical to what they were at the beginning.

The other seven patients developed iatrogenic thyroid disease after the end of the treatment. The median age in this patient group was 8.4 years. Medulloblastoma of the posterior fossa was diagnosed in five of the cases, ependymoma of the posterior fossa in one case, and pineocytoma in one case. All patients received standard radiation therapy according to the histology and location of the tumor. The tumors were radiated at doses ranging from 54 to 68 Gy. This group of patients developed thyroid hormone dysfunction at variable time intervals from the end of treatment. The mean time was 68.57 months, with values ranging from 20 to 123 months. All seven patients were symptomatic when the hypothyroidism was discovered. Two of the patients developed somatotropic hormone deficiency simultaneously with the thyroid dysfunction. The other five patients were diagnosed with hypothyroidism alone.

**Figure 2:**
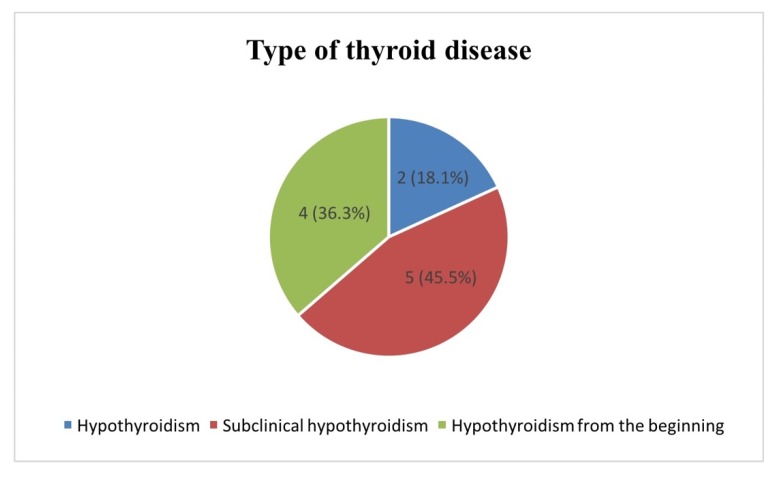
Types of thyroid disorders.

Of the eleven children diagnosed with iatrogenic thyroid disease, two cases were identified as having clinical hypothyroidism, five had subclinical hypothyroidism, and four had hypothyroidism from the beginning of the diagnosis as a complication of the brain tumor (Figure 2).

Over the course of the oncological treatment, all patients underwent biological monitoring as follows: blood count, evaluation of hepatic and renal function, serum electrolytes, and inflammatory tests. They developed treatment-related toxicity, in particular Grade 3 or Grade 4 hematologic toxicity, and mild liver enzyme elevations. At the end of the oncological treatment, all treatment-induced toxicities subsided. During follow-up, the recorded biological values showed no alterations.

All children diagnosed with thyroid dysfunction underwent periodic endocrinologic and anthropometric evaluations. There was one recorded case of delayed puberty. None of the children suffered from height or weight gain deficiencies.

All four cases diagnosed with initial thyroid disease also had combined pituitary hormone deficiency. One of the eleven children had severe permanent neurologic and psychiatric sequelae (Foville syndrome and severe mental retardation).

**Figure 3:**
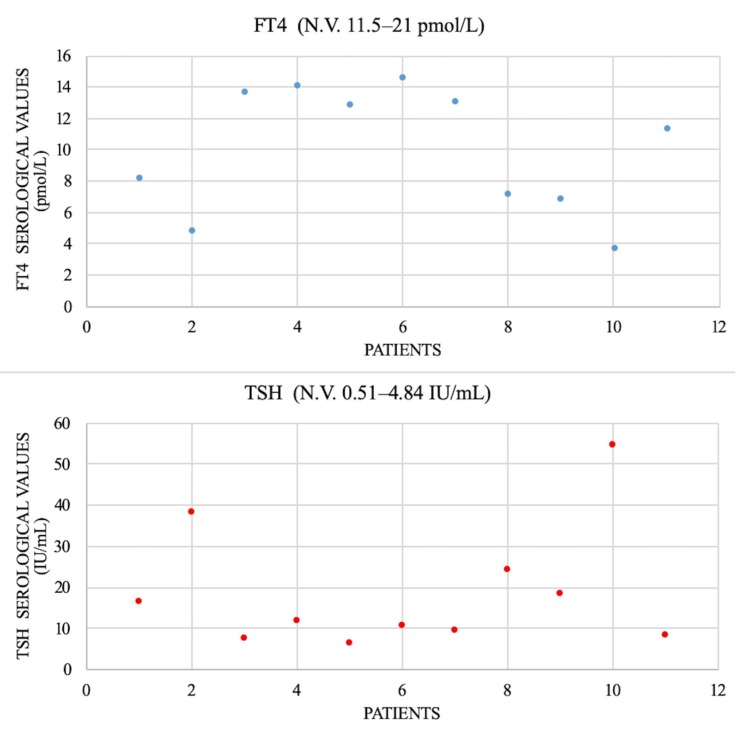
Serological levels of thyroid-stimulating hormone (TSH – red dots) and free thyroxine (FT4 – blue dots) of the patients who developed dysfunction after brain tumor treatment.

All patients underwent serologic thyroid-stimulating hormone (TSH) evaluation (N.V. 0.51–4.84 IU/L) and free thyroxine (FT4) (N.V. 11.5–21 pmol/L); the results are shown in Figure 3. There was no determination of free triiodothyronine (FT3). We checked the thyroid antibodies: antithyroglobulin (anti-Tg, N.V. < 3 months < 146 IU/mL; 1–6 years < 38 IU/mL; 6–11 years < 37 IU/mL; and 11–20 years < 64 IU/mL) and antiperoxidase antibody (anti-TPO, N.V. < 26 IU/mL). We found only two cases with increased levels suggesting chronic lymphatic thyroiditis.

**Figure 4:**
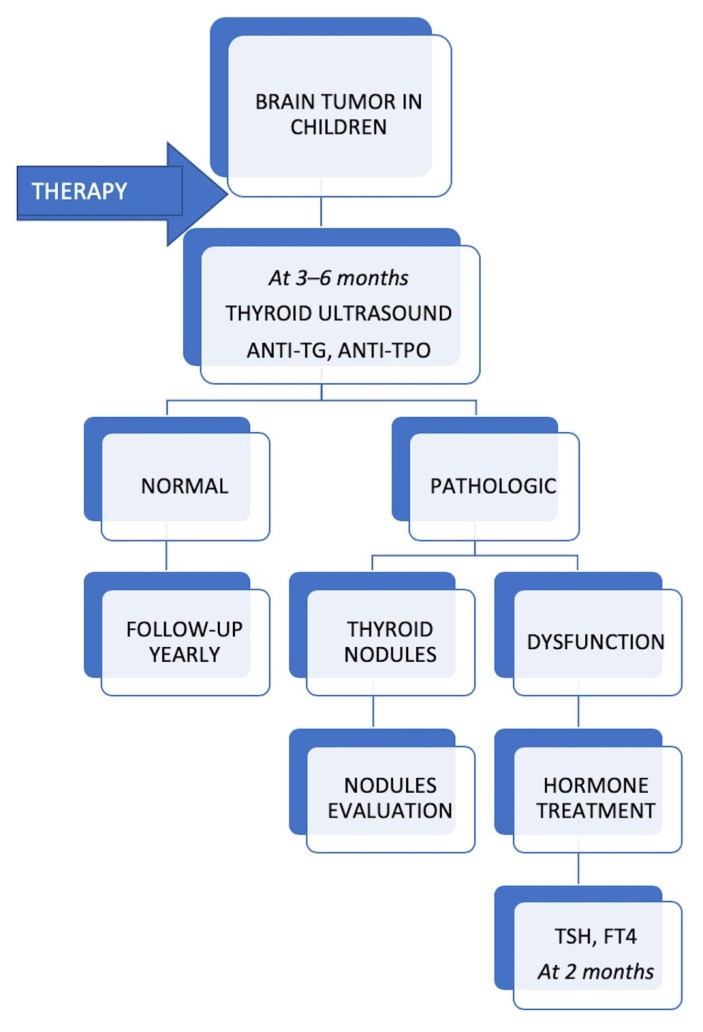
Protocol for monitoring the thyroid gland of children with brain tumors during follow-up.

All patients with thyroid dysfunction had a thyroid ultrasound every 6 months, which showed no thyroid nodule developing during follow-up. We would like to emphasize that no case of thyroid cancer has been registered so far. All patients received hormonal substitution with levothyroxine and achieved normal thyroid status during follow-up. The proposed protocol for monitoring the thyroid function of children with brain tumors during follow-up is shown in Figure 4.

## 4. Discussion

We studied 178 patients with brain tumors, with a median age of 8.4 years. One hundred and seventy-eight patients received standard therapy according to the histology and staging of the disease. Six patients abandoned the therapy. One hundred and fifty-eight children underwent surgery. One hundred and thirty-three of the patients received standard chemotherapy. One hundred and twenty patients received radiation to the craniospinal axis.

Treatment-induced hypothyroidism following radiotherapy in children treated for primary brain tumors is a relatively frequent side effect in this patient group. According to literature, iatrogenic hypothyroidism occurs late after the end of radiation therapy, with a prevalence of 15% [1].

Of the 120 surviving patients, we recorded 11 cases of iatrogenic thyroid disease, accounting for 9.1% of the total. All patients had a minimum follow-up of 4 years.

It is known that patients irradiated for brain tumors with more than 18 Gy are at risk of developing thyroid dysfunction [9]. The children in this study received radiation doses ranging from 40 to 68 Gy.

Some authors report a high prevalence of iatrogenic hypothyroidism in children who have had brain radiation to the posterior fossa [10]. In this study, 6 out of the 11 patients diagnosed with thyroid hormone deficiency had posterior fossa tumors.

Additionally, there is a significantly increased risk for developing endocrinological side effects in children diagnosed with brain tumors located in the proximity of the hypothalamic and pituitary region [11]. In our patient group, 5 of the 11 patients had tumors located in this region. Four patients suffered from combined pituitary hormone deficiency from the beginning and one developed hypothyroidism 4 years after receiving radiotherapy. Two of the children developed growth deceleration, and there was one case of puberty delay.

If left untreated, the clinical significance of impaired thyroid hormone secretion has a great impact on physical and cognitive development. Hormonal deficiencies may be treated successfully with replacement therapy [12,13].

In light of this study, we find that initial endocrinologic evaluation is crucial in all pediatric patients diagnosed with brain neoplasms, especially for those with tumors located in the proximity of the hypothalamic–pituitary axis. This course of action will lead to early diagnosis and treatment of all thyroid deficiencies, thus preventing delays in the physical and cognitive development of the patients.

With further experience and larger patient populations, the intervals at which thyroid testing should be performed in children who undergo radiation of the craniospinal axis will be established. We find that thyroid disease screening should be carried out routinely on all children treated for brain tumors every 6 months for at least 7 years.

It is uncertain whether the patients in this study will develop thyroid neoplasms later in life, as has been demonstrated by some study groups [14].

## 5. Conclusions

Childhood brain tumor survivors represent a distinct group of patients who require lifelong follow-up and management in order to provide optimal development and quality of life. Patients diagnosed with tumors located close to the hypothalamic–pituitary axis are at great risk of developing combined pituitary hormone deficiency, not only as a result of oncological treatment but also as a consequence of tumoral growth. The number of patients diagnosed with iatrogenic thyroid disease in our retrospective cohort is comparable with other studies that looked at the same pathology. In future research, the exact role of radiotherapy in the development of iatrogenic hypothyroidism in larger patient populations should be considered as a secondary objective.

## Figures and Tables

**Table 1:** Radiotherapy dosage.

Diagnosis	Brain Radiation Dosage	Craniospinal Axis Dosage
Medulloblastoma of the posterior fossa, Grade 4	54 Gy	30 Gy
Ependymoma of the posterior fossa, Grade 2	54 Gy	
Medulloblastoma of the posterior fossa, Grade 4	68 Gy	36 Gy
Pilocytic astrocytoma of the optic chiasm, Grade 1	45 Gy	
Hypothalamic germinoma, Grade 4	45 Gy	30.6 Gy
Medulloblastoma of the posterior fossa, Grade 4	45 Gy	27 Gy
Hypothalamic germinoma, Grade 4	40 Gy	30 Gy
Medulloblastoma of the posterior fossa, Grade 4	53.6 Gy	22.4 Gy
Pineocytoma, Grade 2	54 Gy	
Pituitary adenoma	50.4 Gy	
Medulloblastoma of the posterior fossa, Grade 4	55.8 Gy	23.4 Gy
